# Tubulin Tyrosine Ligase Like 4 (TTLL4) overexpression in breast cancer cells is associated with brain metastasis and alters exosome biogenesis

**DOI:** 10.1186/s13046-020-01712-w

**Published:** 2020-09-30

**Authors:** Julia Arnold, Juliana Schattschneider, Christine Blechner, Christoph Krisp, Hartmut Schlüter, Michaela Schweizer, Marcus Nalaskowski, Leticia Oliveira-Ferrer, Sabine Windhorst

**Affiliations:** 1 Department of Biochemistry and Signal Transduction, University Medical Center Hamburg-Eppendorf, Martinistrasse 52, 20246 Hamburg, Germany; 2 Institute of Clinical Chemistry and Laboratory Medicine, Mass Spectrometric Proteomics, University Medical Center Hamburg-Eppendorf, 20246 Hamburg, Germany; 3 Core Facility Morphology und Electron Microscopy, Center for Molecular Neurobiology Hamburg, ZMNH, University Medical Center Hamburg-Eppendorf, Falkenried 94, 20251 Hamburg, Germany; 4 Department of Gynecology, University Medical Center Hamburg-Eppendorf, Martinistrasse 52, 20246 Hamburg, Germany

**Keywords:** Breast cancer, Metastasis, Exosome, Microtubule, Polyglutamylation

## Abstract

**Background:**

The survival rate is poor in breast cancer patients with brain metastases. Thus, new concepts for therapeutic approaches are required. During metastasis, the cytoskeleton of cancer cells is highly dynamic and therefore cytoskeleton-associated proteins are interesting targets for tumour therapy.

**Methods:**

Screening for genes showing a significant correlation with brain metastasis formation was performed based on microarray data from breast cancer patients with long-term follow up information. Validation of the most interesting target was performed by MTT-, Scratch- and Transwell-assay. In addition, intracellular trafficking was analyzed by live-cell imaging for secretory vesicles, early endosomes and multiple vesicular bodies (MVB) generating extracellular vesicles (EVs). EVs were characterized by transmission electron microscopy (TEM), nanoparticle tracking analysis (NTA), Western blotting, mass spectrometry, and ingenuity pathway analysis (IPA). Effect of EVs on the blood-brain-barrier (BBB) was examined by incubating endothelial cells of the BBB (hCMEC/D3) with EVs, and permeability as well as adhesion of breast cancer cells were analyzed. Clinical data of a breast cancer cohort was evaluated by χ2-tests, Kaplan-Meier-Analysis, and log-rank tests while for experimental data Student’s T-test was performed.

**Results:**

Among those genes exhibiting a significant association with cerebral metastasis development, the only gene coding for a cytoskeleton-associated protein was Tubulin Tyrosine Ligase Like 4 (TTLL4). Overexpression of TTLL4 (TTLL4^plus^) in MDA-MB231 and MDA-MB468 breast cancer cells (TTLL4^plus^ cells) significantly increased polyglutamylation of β-tubulin. Moreover, trafficking of secretory vesicles and MVBs was increased in TTLL4^plus^ cells. EVs derived from TTLL4^plus^ cells promote adhesion of MDA-MB231 and MDA-MB468 cells to hCMEC/D3 cells and increase permeability of hCMEC/D3 cell layer.

**Conclusions:**

These data suggest that TTLL4-mediated microtubule polyglutamylation alters exosome homeostasis by regulating trafficking of MVBs. The TTLL4^plus^-derived EVs may provide a pre-metastatic niche for breast cancer cells by manipulating endothelial cells of the BBB.

## Background

Breast cancer brain metastasis is associated with poor prognosis and 10 to 30% of breast cancer patients develop brain metastases. Breast cancer cells that preferentially metastasize to the brain mostly belong to the subgroup of Her2 overexpressing cells or triple-negative breast cancer cells (TNBC). These cancer cells are highly aggressive and since most therapeutic compounds do not pass the blood-brain-barrier (BBB), they do not respond to hormone or chemotherapy. Clinical trials for new targeted therapies are in progress and the preliminary results are promising [1–3].

During adhesion on and transmigration through the endothelial cell layer of the BBB, the cytoskeleton of cancer cells must be tightly controlled [4]. Therefore, proteins regulating cytoskeletal functions are interesting targets for prevention of cancer cell penetration into the brain. The cytoskeleton mainly consists of actin filaments (F-actin), microtubules (MTs) and intermediate filaments, which can interact with each other. F-actin is essential for the formation of cellular protrusions involved in adhesion, chemotaxis (filopodia), migration (lamellipodia) and invasion (invadopodia). These actin-rich structures are dynamically formed and degraded, requiring precise recruitment and activation of actin-binding proteins (reviewed in [5]).

Yet, the largest compartment of the cytoskeleton is established by the dynamic network of MTs, composed by α- and β-tubulin dimers forming cylindrical polymers. MTs fulfil various functions like motility in cilia or flagella, the formation of the mitotic spindle as well as intracellular transport. These functions can be directed by differential expression of tubulin isoforms and a plethora of posttranslational modifications (PTMs), acting as a rheostat.

These PTMs are mediated by enzymes inducing and reversing acetylation, tyrosination, polyglycylation as well as polyglutamylation. Hence, PTMs coexists and might interact on the same MT. Decoration of the MT-surface with PTMs results in a wide and fine-tuned range of effects on interacting proteins. The composed “tubulin code” is yet beginning to unravel (reviewed in [6, 7]). The PTM polyglutamylation enhances the number of negative charges and changes the conformation on the MT-surface, which affects the interaction with MAPs and motor proteins [7–9]. Glutamylation of MTs is executed by Tubulin Tyrosine Ligase Like (TTLL) enzymes. The addition of the initial glutamate (Glu) residue to the γ-carboxyl group near the C-terminus of polymerized tubulin can be catalyzed by TTLL4 [10–13]. TTLL4 prefers β-tubulin and acts as an “initiase” or “monoglutamylase”, creating short Glu chains [14], which can be further elongated by “polyglutamylases” e.g. TTLL1 [15–22].

Affinity of vesicle transporting kinesins to MTs depends on the length of Glu chains [19]. Cellular vesicles include endosomes, which can generate intraluminal vesicles (ILV) by invagination and result as MVB, presenting Rab7. Then, late endosomes/MVBs can be transported along MTs to the plasma membrane, where they fuse with the plasma membrane to release the ILV as exosomes [20]. By different loading and sorting mechanism exosomes include proteins, micro ribonucleic acids (miRNA), non-coding ribonucleic acids (ncRNA) and DNA. Their size typically ranges within 50 nm to 150 nm [21]. Since they can be internalized by neighboring or distant cells, secretion of exosomes by tumour cells primes their invasion and/or the tumour microenvironment to survive at distant sites [22, 23].

Here, we analyzed the expression of significantly deregulated genes in primary breast cancer cells from patients with brain metastasis. Among these, TTLL4 was the only cytoskeleton-associated protein whose increased expression correlated with brain metastasis formation. Based on this finding, in this study we analyzed the functional role of TTLL4 overexpression for TNBC.

## Methods

### Microarray data

Microarray analyses of TTLL4 messenger RNA (mRNA) levels were analysed in a cohort of 197 primary breast cancer tissue samples [24, 25]. All patients were treated between 1991 and 2002 and were selected based on tissue availability. No radiotherapy, neoadjuvant chemotherapy or endocrine therapy had been administered before surgery. All patients gave written approval for the utilisation of their tissue samples and the reviewing of their medical records according to our investigational review board and ethics committee guidelines (Ethik-Kommission der Ärztekammer Hamburg, #OB/V/03). We analyzed the TTLL4 mRNA level (probeset 203702_s_at) using microarray data (Affymetrix HG-U133A) from the cohort mentioned before. The cohort was firstly divided into quartiles of similar size, representing low, moderate-low, moderate-high, and high TTLL4 levels. Since cases with low, moderate-low and moderate-high TTLL4 mRNA levels behaved similar in survival analysis, we combined these groups (TTLL4 level < 75%) for further analysis (Figure S1A, B). Correlations between TTLL4 mRNA levels (cut-off 75% percentile) and clinicopathological factors such as histological grading, stage, lymph node involvement, estrogen and progesterone receptor status (ER, PR), molecular subtype and metastasis formation were statistically examined by χ2-tests. Overall and recurrence-free survival was analysed by Kaplan-Meier analysis and Log-Rank-Tests. All statistical analyses were conducted using SPSS software Version 26 (SPSS Inc., Chicago, IL, USA).

### Overexpression of TTLL4 in MDA-MB231 and MDA-MB468 cells

The complementary DNA (cDNA) for TTLL4 was purchased from Source BioScience (ORFeomeV8.1 #CCSBo5058H0981131D) and was cloned into the Lego-iC2/Puro+ vector which was a friendly gift from Dr. Kristoffer Riecken (Department of Stem Cell Transplantation, University Medical Center Hamburg-Eppendorf (UKE)). Virus production, infection of MDA-MB231 and MDA-MB468 cells, and selection with puromycin were performed as described [26]. Overexpression was verified by real-time polymerase chain reaction (rt-PCR).

### Cell culture

All cells were cultured in T75 tissue culture (TC) flasks (Sarstedt #83.3911.002) and 15 cm TC dishes (Sarstedt #83.3903) at 37 °C, 5% CO_2_, 100% humidity. MDA-MB231 (ATCC #HTB-26) and MDA-MB468 (ATCC #HTB-132) cells required DMEM culture media (Gibco, #41965–035) containing constantly 10% fetal calf serum (FCS) (Gibco), penicillin-streptomycin (100 U/ml) (Gibco) and puromycin (2 μg/ml) (Gibco).

hCMEC/D3 cells (Merck, #SCC066) were grown in EBM-2 medium (Lonza #CC-3156) containing, 5% FCS (Gibco), 1x chemically defined lipid concentrate (Gibco #11905–031), 5 μg/ml ascorbic acid (Sigma #A4544) and 1.4 μM hydrocortisone (Sigma # H0888).

### Immunocytochemistry and Western blotting

For immunocytochemistry, cells grown on chamber slides (μ-Slider 8-well, Ibidi, Munich, Germany, #80826) were fixed with 4% paraformaldehyde/4% sucrose/PBS for 15 min, permeabilized with 0.5% TritonX-100/PBS for 5 min, both at room temperature (RT) and blocked with 1 x PBS/0.05% TritonX-100, containing 5% bovine serum albumin (BSA) for 20 min at RT. The primary antibodies were diluted 1:200 in blocking solution and incubated over-night at 4 °C. Primary antibodies used were anti-ß-tubulin (Santa Cruz #SC-9104), anti-NAP-1 (abcam #ab21630), anti-polyglutamylation modification GT335 (AdipoGen, #AG-20B-0020-C100), anti-Rab7 (Sigma-Aldrich #R8779) and anti-VASP (Santa Cruz #SC-46668). After washing with PBS/0.03% TritonX-100, the secondary Alexa-Fluor (AF) coupled antibodies against rabbit AF-568 (abcam #ab175695) or mouse AF-488 (Life technologies Eugene #A21202) or mouse AF-568 (Life technologies Eugene #A11031) were applied for 1 h at RT in a dilution of 1:2000 in blocking solution. Thereafter, the cells were stained with 0.2 μg/ml of 4′,6-diamidino-2-phenylindole solution (DAPI; Roth #6335) in PBS for 5 min at RT and/or with AF-488 coupled phalloidin (Thermo Fisher #A12379) in a dilution of 1:1000 at RT for 45 min. Finally, the cells were washed 3-times with PBS.

Images were acquired using Leica TCS SP8 X with a 63x NA = 1.4 oil objective lens controlled by Leica LAS X. Samples stained with AF-488 coupled phalloidin or with an AF-488 coupled antibody against VASP were imaged using Keyence BZ1000.

Western blotting was performed according to standard protocol using the mentioned primary antibodies and secondary anti-rabbit (Santa Cruz #SC-2054) or anti-mouse (Santa Cruz #SC-2055) antibodies in dilution of 1:5000. Bands were detected by bioluminescence using ECL Prime Western Blotting Detection Reagent (Amersham #RPN2232) in an ImageQuant LAS4000 System (GE Healthcare).

### Immunoprecipitation

Cells were seeded to 15 cm TC-dishes and after grown to 90% confluence, they were washed twice with ice-cold PBS. Thereafter, 1 ml M-PER™ buffer (ThermoFisher #78501) including protease inhibitor cocktail (cOmplete™, EDTA-free protease inhibitor cocktail, Roche #04693132001) were added, cells were scraped and stored at − 80 °C. To couple the beads with antibody, 50 μl Protein A sepharose (abcam #ab193256) was incubated with 5 μl GT335 antibody (1 mg/ml, see above) for 3.5 h at 17 °C under rotation. In between the cell lysates were thawed, vortexed vigorously and centrifuged at 13.000 g at 4 °C. The supernatant was transferred to GT335-coupled Protein A sepharose beads, which had been equilibrated twice with M-PER™ buffer. After incubating the beads for 4 h at 4 °C under rotation, the supernatant was removed, and the beads were washed thrice with M-PER™ buffer. Finally, 2 x SDS sample buffer was added, the beads were incubated for 7 min at 95 °C, centrifuged for 1 min at 13.000 g to remove bound proteins and protein concentrations were analysed by Western blotting.

### Adhesion assay

To analyze adhesion of breast cancer cells to hCMEC/D3-cells, 2 × 10^4^ hCMEC/D3-cells were seeded to chamber slides (see above) and grown for 5 days. Confluency of the cells was controlled by staining parallel cells with AF-488 labeled phalloidin (see above). Then, 5000 CellTracker™ Green CMFDA (Invitrogen #C2925)-loaded breast cancer cells were seeded onto confluent hCMEC/D3-cells. After incubation for 4 h, the fluorescence of breast cancer cells was analyzed with the Keyence BZ1000 microscope and cell number determined by its cell count function.

### Migration

1 × 10^5^ MDA-MB231 cells were seeded to 96-well plates and grown for 2–3 days until confluence. Migration was examined by inserting a scratch into a confluent MDA-MB231 cell layer and migration into the wound was measured by IncuCyte® Live-Cell Analysis system (Sartorius) and quantified by using the software IncuCyte Zoom® (Version 2016B).

### Transmigration

Fifty thousand cells were seeded into Boyden chambers (Costar #3422), containing medium with 10% in the upper and 10% in the lower chamber. An FCS gradient created by adding different FCS concentration in the upper and the lower chamber disappears after about 5 h (own preliminary studies). Since adhesion to and migrations through the Boyden chamber of tumour cells is not fast enough to profit from this gradient, here the FCS concentrations were equal between the upper and the lower chamber. After seeding, the cells were incubated for 48 h, cells of the upper site of the membranes were washed away with a cotton swab, the membranes were sliced, cells were stained with DAPI and counted, using the cell count function from Keyence BZ1000 microscope.

### Viability

Viability was measured by a standard MTT-assay using Methylthiazolyldiphenyl-tetrazolium bromide (MTT, Sigma-Aldrich #M2128) and Dimethylsulfoxide (DMSO, Sigma-Aldrich #D4540). Subsequent detection was performed after 24 h, 48 h and 72 h by a plate reader at 350 nm absorption.

### EV isolation

Conditioned media of breast cancer cells grown in DMEM/10% FCS were collected after 4 days of incubation under standard conditions, and then vesicles were purified by differential centrifugation at 4 °C. First the sample was centrifuged at 500 g for 10 min to eliminate detached cells, followed by centrifugation at 10,000 g for 30 min to pellet cell debris. Finally centrifugation at 100,000 g for 90 min was performed to harvest EVs. The exosome pellet was washed for cellular assays with 1 x PBS pH 7.4 or with 1 mM HEPES pH 7.4, containing EDTA-free Protease Inhibitor Cocktail tablets (see above) for validation and characterization. To assess the biological effects of EVs, the protein content of EVs was analyzed by the Bradford assay and the number of EVs was adjusted to 50 μg protein in total. These values were further normalized to the number of cells where the EVs were harvested from.

### EV validation by transmission electron microscopy (TEM)

Fixation and negative stain for transmission electron microscopy (TEM) were conducted as previously described [27]. In brief, Formvar (EMS, Hatfield USA, #15800) coated copper electron microscopy (EM) grids (Plano, Wetzlar Germany, #G2410C-X) were incubated for 10 min on drops of 0.01% poly-L-lysine hydrobromide (Sigma, St Louis USA, #P2636-100MG) for hydrophilicity and stickiness. Grids were then washed over four droplets on doubled-distilled water and the excess water was removed from the grids using filter paper. Freshly isolated EVs were resuspended in 2% paraformaldehyde (PFA) in PBS (Carl Roth, Karlsruhe #0964.4) before the grids were placed with the hydrophilic surface down onto the droplet. After incubation for 20 min, the grids were washed again on five droplets of PBS for 2 min each. Next, a 5 min incubation with 1% glutaraldehyde (GA) in 0.1 M sodium phosphate buffer, pH 7.4 was performed (EMS, Hatfield USA, #16320). Fixation was finished after washing over seven droplets of double-distilled water for 2 min each. For negative staining, grids were transferred to the uranyl-oxalate solution (pH 7), (EMS, Hatfield USA, #22400) for 5 min before further incubation with methylcellulose-uranyl-acetate (Sigma, St Louis USA, #M-6385) for 10 min on ice. Finally, the grids were dried using a filter paper. Images were acquired with the electron microscope (EM 900; Zeiss) at 80 kV equipped with a 4 K digital camera (Tröndle, Moorenweis, Germany).

### EV characterization by nanoparticle tracking analysis (NTA) and mass spectrometry

For NTA, samples were measured by using the NanoSight LM14 (NanoSight NTA 2.3 Build 0033, Malvern Instruments) employing a 638 nm laser and a Marlin F-033B IRF camera (Allied Vision Technology). Quantification and characterization were performed by five videos of 60 s length each at a camera intensity of 13 in the batch processing function.

Liquid chromatography-mass spectrometry/mass spectrometry (LC-MS/MS), sample preparation, measurements and data processing are described in detail in Supplementary methods. The label-free quantification (LFQ) intensity values for protein groups were used as main columns. The quantitative values for all protein groups were transformed into log2 values and normalized by the median. This dataset of EV proteome is included within the additional files. Hierarchical clustering, Student’s T-Test, principal component analysis and ingenuity pathway analysis (IPA) were performed.

### Live-cell imaging of vesicle trafficking

For analysis of green fluorescent protein (GFP)-BDNF vesicle trafficking, 2.5 × 10^4^ cells were seeded to chamber slides (see above). Next day the cells were transfected with a vector encoding for GFP-BDNF (a friendly gift from Prof. Dr. Matthias Kneussel, Center for Molecular Neurobiology, UKE) using K2® Transfection System (Biontex #T060) according to the manufacturer’s instructions. After 48 h of incubation, the movement of vesicles was monitored every 5 s by live fluorescence-microscopy (Keyence BZ1000) and tracked manually. Finally, the vesicle speed (μm/s) was calculated.

To analyze trafficking of Rab5 and Rab7 vesicles, 1 × 10^6^ cells were seeded on 18 mm coverslips and transfected the next day as described above. Vectors coding for monomeric red fluorescent protein linked to Ras-related protein (mRFP-Rab5, mRFP-Rab7) were a gift from Ari Helenius (Addgene plasmid # 14437; http://n2t.net/addgene:14437; RRID: Addgene_14,437 and Addgene plasmid # 14436; http://n2t.net/addgene:14436; RRID: Addgene_14,436) [28].

Spinning-disk confocal microscopy was performed with a Nikon Eclipse Ti-E equipped with a 100 x objective (Nikon, ApoTIRF 100×/1.49oil). Analysis of mean speed was done using TrackMate Plugin for FIJI [29].

### Measurement of cell permeability after EV-incubation

2 × 10^5^ hCMEC/D3-cells were seeded to Transwell cell culture chambers with a mean pore size of 0.4 μm (Corning #3470) and incubated for 5 days to reach confluency. Thereafter, EVs (see EV isolation) were applied to hCMEC/D3-cells and incubation was continued for 16 h. Permeability of hCMEC/D3-cells was measured by exchanging the cell culture medium to 150 μl medium without phenol red (Gibco #21063029), including 25 μl fluorescein isothiocyanate-dextran (40,000 MW, 6 mg/ml, Sigma FD40). After incubation for 4 h, fluorescence of the medium in the lower reservoir of the 24-well plate was measured in a plate reader at 485 nm excitation and 535 nm emission.

### Statistical analysis

Statistical analysis of clinical data was conducted using SPSS software Version 24 (IBM SPSS Statistics, Armonk, NY, USA). χ2-tests were used to examine the correlations between TTLL4 mRNA levels and clinicopathological factors. For prognostic parameters, the following groups were compared: histological grading (G1/2 vs. G3); nodal status (positive vs negative); ER and PR status (positive vs. negative); the presence of bone, lung, visceral or brain metastasis (positive vs. negative); and molecular subtype (luminal vs. human epidermal growth factor receptor 2 (HER2)-positive vs. triple-negative (TNBC)). Survival curves were plotted by Kaplan–Meier analysis. Differences between survival curves were evaluated by log-rank tests. Probability values (p) less than 0.05 were regarded as statistically significant. All other experiments were performed at least three times, normalized to the mean of the control group and visualized by mean ± standard deviation (SD). Using Student’s T*-*Tests in GraphPad PRISM 8.0 (GraphPad Software Inc., San Diego, USA) results were considered significant when *p* < 0.05.

## Results

### TTLL4 mRNA levels significantly correlate with shorter recurrence-free survival and brain metastasis in breast cancer patients

To assess a potential relevance of cytoskeletal dynamics in breast cancer progression, we analyzed mRNA expression of genes significantly deregulated in breast cancer samples from patients (*n* = 197) that developed brain metastasis. Characteristics of the patient cohort have been described in the methods section. Among these, TTLL4 was the only cytoskeleton-associated protein whose up-regulated expression significantly correlated with cerebral metastasis formation.

**Fig. 1:**
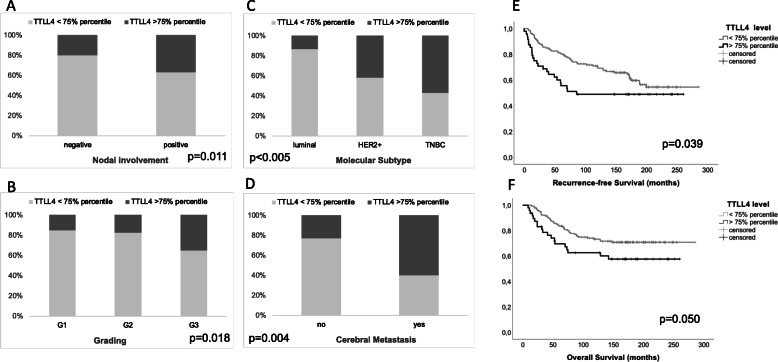
TTLL4 mRNA levels in breast cancer tumour tissue. **a-e** Correlation of TTLL4 mRNA levels with clinical and histological parameters. Significant associations of high TTLL4 levels with positive nodal status (**a**), higher grading (**b**), HER2+ and TNBC subtypes (**c**) and brain metastasis formation (**d**) are shown. Additionally, Kaplan-Meier analysis shows a correlation between high TTLL4 mRNA levels with shorter recurrence-free (**e**) and overall survival (**f**). *P*-values after log-rank tests comparing two groups (TTLL4 levels < 75% vs. TTLL4 levels > 75%) are shown

The mRNA levels of TTLL4 (probeset 203702_s_at) ranged from 8.9 to 1645.5 (mean:119.4 and median:199.1). According to these values, the cohort was first divided into four groups (quartiles) of the same size displaying low, moderate-low, moderate-high and high TTLL4 expression. In survival analysis, we observed that quartiles 1 to 3 behaved similarly and were grouped for further analysis. This new group was defined as “TTLL4 < 75% percentile” and was compared with the remaining group with TTLL4 levels > 75% percentile (Figure S1A, B). Using this cut-off, statistical correlations of TTLL4 levels and clinical and pathological data were performed. Here, high TTLL4 levels (> 75% percentile) were significantly associated with a positive nodal status (Fig. 1a, *p* = 0.011) and higher grading (Fig. 1b, *p* = 0.018). In comparison with luminal breast cancer patients, tumours from HER2+ and triple-negative BC patients showed significantly higher TTLL4 mRNA levels (Fig. 1c, *p* < 0.005). Correspondingly, a strong correlation of high TTLL4 levels and the lack of estrogen and progesterone receptor in tumour tissue was found (Figure S1C, D, *p* < 0.001 and *p* = 0.001, respectively). In the majority of cases showing tumour recurrence, the site of metastasis is documented in our cohort. Interestingly, primary tumours that later developed brain (Fig. 1d) and lung metastasis (Figure S1E) were characterized by higher TTLL4 levels compared with tumours without and these differences were statistically significant (*p* = 0.004 and *p* = 0.02, respectively) (Figs. 1d, S1E). Further, predictive value for TTLL4 was found, namely high TTLL4 levels were significantly associated with shorter recurrence-free survival in Kaplan-Meier analysis (Fig. 1e, *p* = 0.039). The correlation between TTLL4 expression and the overall survival of breast cancer patients showed the same trend and was nearly significant (Fig. 1f, *p* = 0.05).

### TTLL4-overexpression increases MT-polyglutamylation in breast cancer cells

**Fig. 2:**
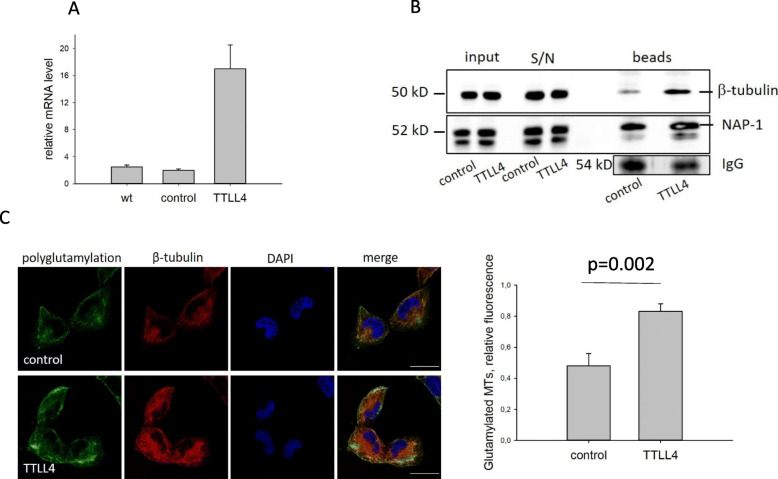
Overexpression of TTLL4 in MDA-MB231 cells increases MT-glutamylation. **a** TTLL4 was overexpressed by using a lentiviral vector. Success of overexpression was analyzed by real-time PCR. Shown are mean values ± SD of three different experiments. Wt = untreated control cells, control = cells treated with Lego vector, TTLL4 = cells treated with Lego vector encoding for TTLL4. **b** Polyglutamylated proteins were immunoprecipitated from control and TTLL4^plus^ cells, using an antibody against polyglutamylation modification (GT335). Cell lysates (“input”), supernatants (“S/N”) from cell lysates incubated with GT335-coupled beads and proteins bound to GT335 coupled beads (“beads”) were analyzed by Western blotting for ß-tubulin and NAP-1 levels. IgG signals served as loading control. The lower band appearing in the NAP-1 blot is a residue signal of β-tubulin antibody because the same membrane was used to probe against all 3 proteins. **c** Fixed cells were labeled using the GT335 antibody (green), ß-tubulin antibody (red) and DAPI (blue) to mark nuclei. Bar: 20 μm. Fluorescence was analyzed by confocal microscopy. Right panel: Fluorescence of GT335 and ß-tubulin signals were analyzed and the ratio GT335/ß-tubulin (Glutamyated MTs) was calculated. Shown are mean values ± SD of 40 cells

To analyze the functional role of TTLL4 in breast cancer cells, stable overexpression of TTLL4 (TTLL4^plus^) was conducted in a TNBC cell line with comparably low endogenous expression (MDA-MB231, see Figure S2) by a lentiviral approach. Real-time PCR analysis of control and TTLL4^plus^ cells revealed a 16-fold increase of TTLL4 mRNA levels in TTLL4 overexpressing cells (Fig. 2a). Since TTLL4 catalysis the first step in polyglutamylation of proteins, this PTM should be increased in TTLL4^plus^ cells. To analyze this assumption, polyglutamylated proteins were immunoprecipitated using the GT335 antibody. Known substrates of TTLL4 are β-tubulin and NAP-1 [30, 31]. Therefore, the GT335 beads were probed against β-tubulin and NAP-1 (Fig. 2b). Evaluation of band intensity normalized to the IgG signals revealed a 2.5-fold or a 1.5-fold increased polyglutamylation of β-tubulin or NAP-1, respectively. Thus, increased expression of TTLL4 in MDA-MB231 cells mainly elevated the level of polyglutamylated β-tubulin.

To confirm increased polyglutamylation of β-tubulin in TTLL4^plus^ cells, fixed cells were stained for polyglutamylation (green), β-tubulin (red) and DNA (DAPI, blue). Fluorescence signals derived from polyglutamylated MTs and β-tubulin were analyzed by confocal fluorescence microscopy, evaluated by ImageJ and normalized to β-tubulin signals (Fig. 2c, right panel). Again, this result shows a clear increase in polyglutamylated MTs in TTLL4^plus^ compared to control cells.

Because of known MT-actin crosstalk in cells [4], we next examined whether increased polyglutamylation of β-tubulin may affect actin dynamics. For this, the F-actin concentration of phalloidin-stained cells (Figure S3A) and the number of actin-based cellular protrusion were counted (Figure S3B, C). However, neither the F-actin concentration nor the length of cellular protrusions was different between control and TTLL4^plus^ cells. Thus, it seems that TTLL4 overexpression does not alter the crosstalk between MTs and actin.

In summary, our data show that in control MDA-MB231 cells NAP-1 is highly polyglutamylated and TTLL4 overexpression only slightly increased this level. On the other hand, the level of β-tubulin polyglutamylation is comparably low in control cells and strongly increased after TTLL4 overexpression.

### TTLL4 overexpression accelerates velocity of secretory vesicles and MVBs but has no significant effect on cell viability and migration

**Fig. 3:**
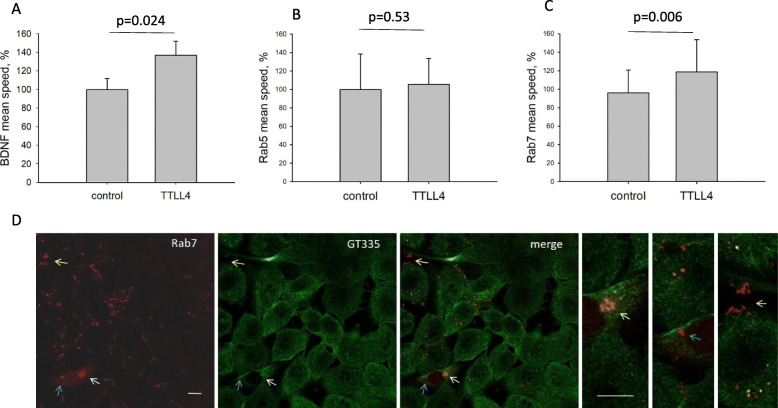
TTLL4 increases vesicle trafficking. **a-c** MDA-MB231 cells were transfected with vectors encoding for GFP-BDNF, mRFP-Rab5 or mRFP-Rab7 and vesicle trafficking (μm/sec) was analyzed by life cell imaging. Shown are mean values ± SD of three different experiments. **d** Control cells (including the Lego vector) were transfected with mRFP-Rab7 vectors, fixed after 24 h of incubation, stained by antibodies against polyglutamylation modification (GT335, green) and analyzed by confocal microscopy. Shown are representative images. Bar: 10 μm. White arrow: Rab7 vesicles colocalizing with polyglutamylated MTs, blue arrow: Rab7 vesicles non- colocalizing with polyglutamylated MTs. Yellow arrow: EVs

**Fig. 4:**
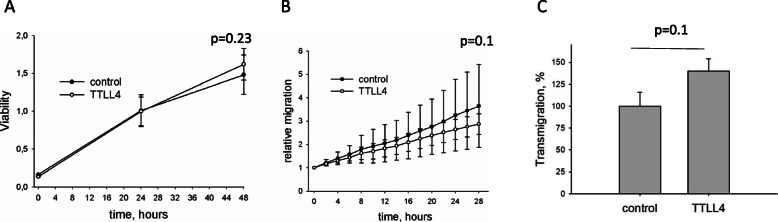
Effect of TTLL4 on the metastatic potential of MDA-MB231 cells in vitro*.* **a** Cell viability was measured by the MTT assay. **b** A scratch was made into confluent MDA-MB231 cells and migration into the wound was analyzed with the IncuCyte® Live-Cell Analysis system (Sartorius) linked to IncuCyte Zoom® (Version 2016B). **c** Transmigration of cells was assessed by the Transwell assay. Shown are mean ± SD of three independent experiments

Polyglutamylation of β-tubulin can alter the affinity of kinesins to MTs and thereby trafficking of cellular vesicles [15–18, 32]. Therefore, it was likely that increased β-tubulin polyglutamylation alters vesicle trafficking. To analyze this assumption, we assessed the speed of secretory vesicles, early endosomes and late endosomes/MVBs. For this purpose, the cells were transfected with vectors coding for GFP-BDNF (brain-derived neurotrophic factor as a marker for secretory vesicles), mRFP-Rab5 (early endosomes) or mRFP-Rab7 (late endosome/MVBs). Vesicle trafficking was analyzed by live-cell imaging. In TTLL4^plus^ cells, the speed of secretory vesicles increased by 38% and the speed of late endosomes/MVBs by 23% (Fig. 3a, c). However, the velocity of early endosomes was unaffected (Fig. 3b). To examine if Rab7-labeled vesicles are transported via polyglutamylated MTs, mRFP-Rab7-transfected MDA-MB231 cells were stained for polyglutamylation (green). Merged channels show that Rab7-labeled vesicles accumulated on polyglutamylated MTs (Fig. 4d, white arrow). However, intracellular vesicles that did not colocalize with polyglutamylated MTs were also detected (Fig. 4d, blue arrow) and Rab7 labeled EVs were found (Fig. 4d, yellow arrow).

These results suggest that high TTLL4 levels promote the transport of particular late endosomes/MVBs by increasing MT-polyglutamylation.

In order to show whether, in addition to altering vesicle trafficking, TTLL4 may affect viability and migration, these cellular processes were analyzed. As shown in Fig. 4a, there was no effect on cell viability (*p* = 0.23) but a tendency that planar migration, measured by the Scratch assay, was reduced. Also, transmigration was increased in TTLL4^plus^ compared to control cells (*p* = 0.1, Fig. 3b, c). However, due to high variability of the cellular migratory capacity, these results were not significant.

In summary, our results show that TTLL4 overexpression increases trafficking of specific vesicles but has no significant effect on viability and migration of MDA-MB231 cells.

### TTLL4 alters EV homeostasis

Our data show that TTLL4 overexpression increases velocity of secretory vesicles as well as of Rab7 containing late endosomes/MVBs. Since MVBs mediate secretion of exosomes/EVs, who are known to condition metastatic niches [33], we next analyzed a potential involvement of TTLL4 on exosomes/EV signature. Possible alteration of exosomes/EV signature could provide a link between the clinical relevance of TTLL4 (Fig. 1) and its effect on MT-polyglutamylation (Fig. 2).

**Fig. 5:**
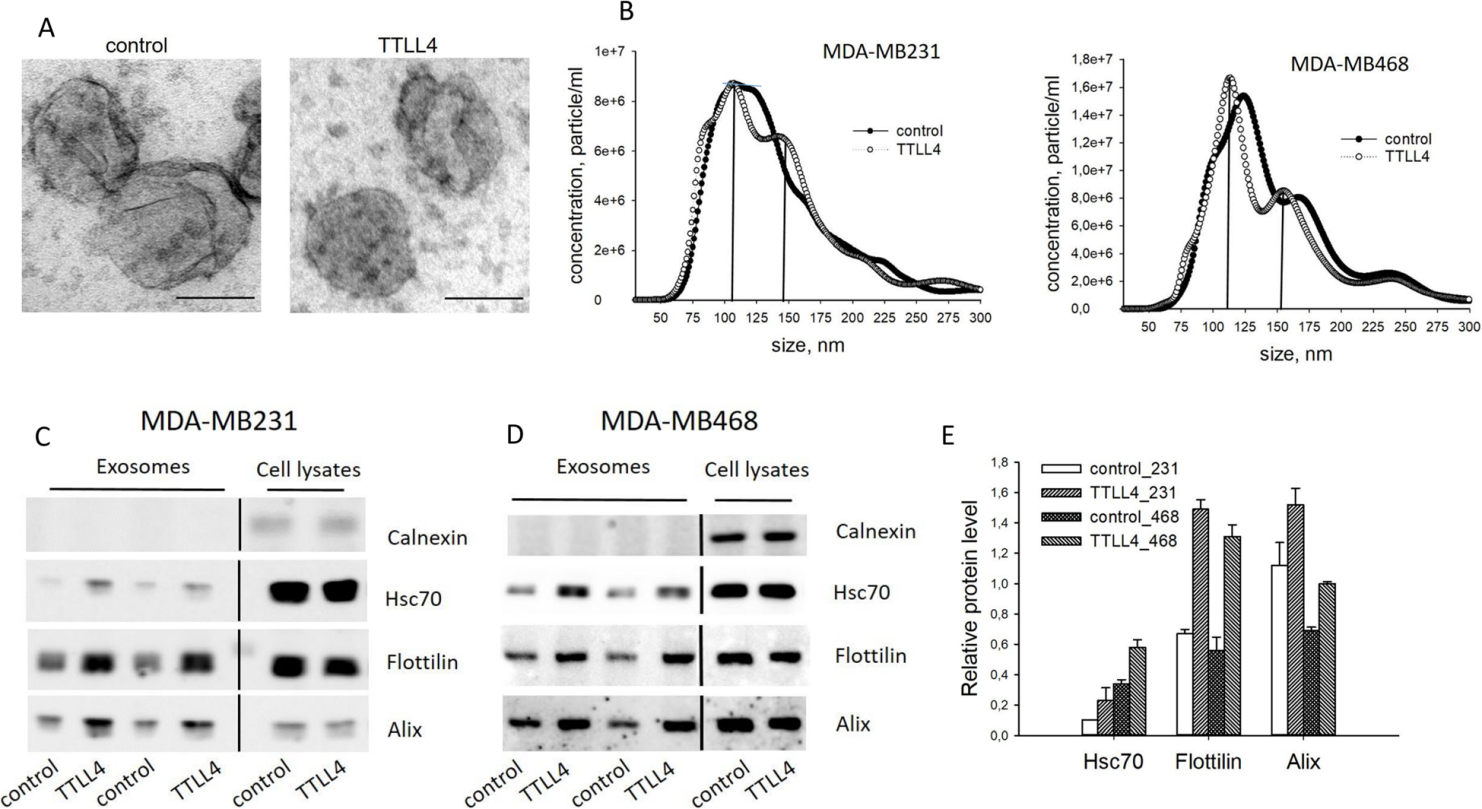
Characterization of EVs from TTLL4^plus^ and control cells. **a** EVs were isolated by differential centrifugation and analyzed by TEM. Shown are representative images, Bar: 100 nm. **b** Particle concentration and size distribution were examined by NTA. Mean of three independent experiments is shown. **c**, **d** The concentration of Calnexin, Hsc70, Flottilin-1 and Alix in EVs were analyzed by Western blotting. **e** Quantification of Hsc70, Flottilin-1 and Alix signals in cell lysates and EVs was performed by ImageJ and ratios of signals derived from EV proteins and cell lysates were calculated. Shown are mean ± SD of four different signals

To address this, EVs (containing exosomes, microvesicles and apoptotic bodies [33]) isolated from media of MDA-MB231 control and TTLL4^plus^ cells were validated by transmission electron microscopy (TEM) visualizing the typical cup shape of exosomes [27] (Fig. 5a). Also, nanoparticle trafficking analysis (NTA) was performed to analyze the concentration and size distribution of EVs. This analysis revealed differences in size-distribution between EVs from control and TTLL4^plus^ cells, while the EV concentration was similar. In control EVs, one non-sharp peak between 80 nm and 125 nm and in TTLL4^plus^ derived EVs two sharp peaks at 110 and 145 nm were observed.

This result indicates that TTLL4 overexpression alters secretion of EV populations.

To validate this finding, TTLL4 was overexpressed in a second TNBC cell line (MDA-MB468), exhibiting slightly higher endogenous TTLL4 mRNA levels compared to MDA-MB231 (Figure S4A). Overexpression of TTLL4 was verified by real-time PCR (Figure S4B) and polyglutamylation level by Western blotting (Figure S4C).

Thereafter, EVs from the medium of MDA-MB468 cells were analyzed by NTA. Size distribution of EVs derived from control cells were different from those of control MDA-MB231 cells, they had two sharp peaks at 125 nm and 170 nm. However, sizes of EVs from both TTLL4^plus^ cell lines were similar. Thus, it seems that TTLL4 overexpression indeed alters biogenesis of EVs.

To validate this conclusion, proteins were extracted from EVs and the concentration of the EV marker proteins Alix, Flottilin-1 and Hsc70 were analyzed by Western blotting. Protein concentration was normalized to cell number and Calnexin served as a negative control. Indeed, we found an increased level of EV markers in both TTLL4 overexpressing TNBC cell lines, ranging from 1.4-fold to 2.3-fold (Fig. 5c-e). Since the protein concentration was not significantly different between EVs from control or TTLL4^plus^ cells (Figure S5A), increased concentrations of Alix, Flottilin-1 and Hsc70 in EVs from TTLL4^plus^ cells further support our conclusion that the EV population secreted by TTLL4^plus^ cells is different from those of control cells.

**Fig. 6:**
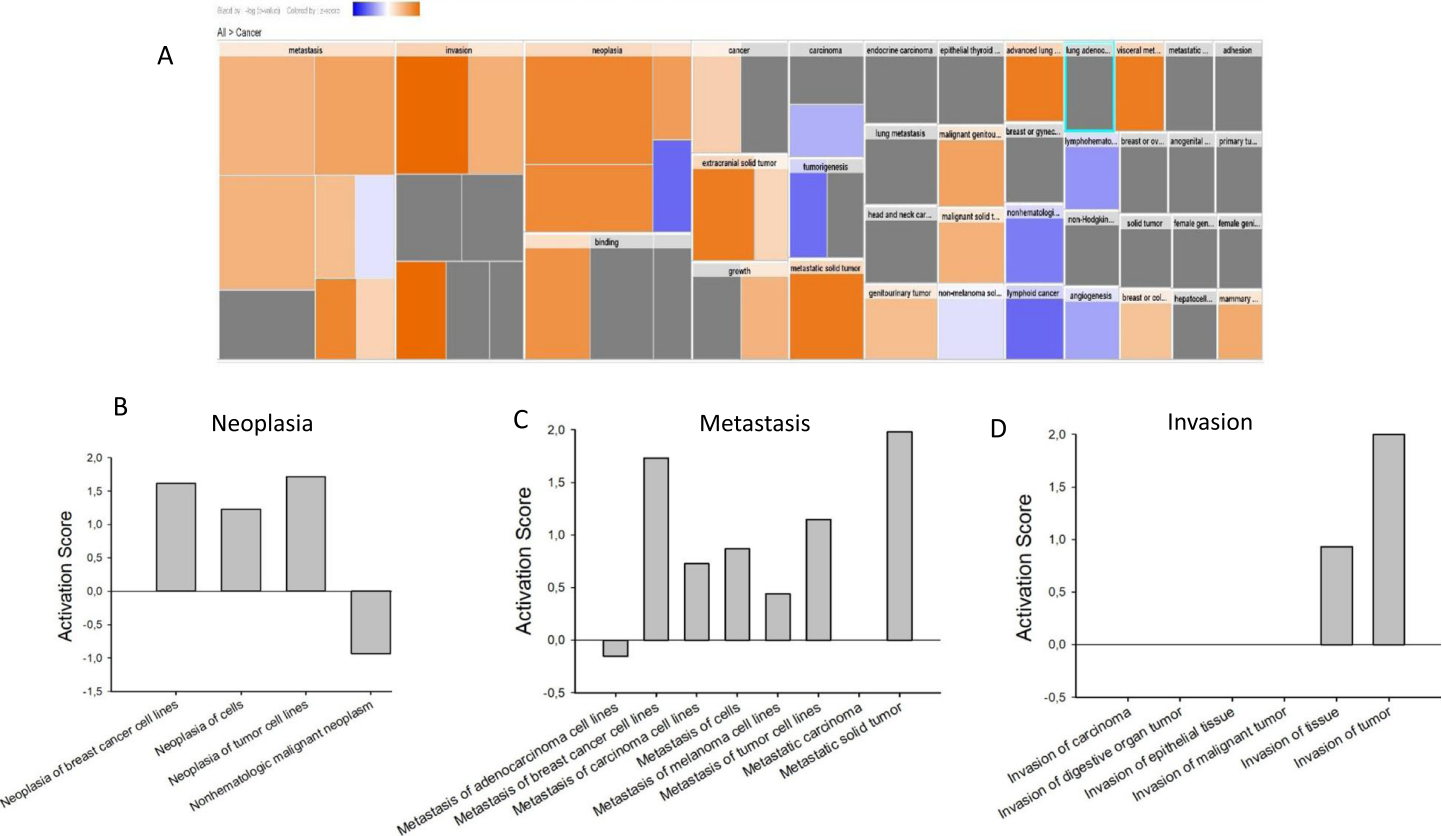
Ingenuity pathway analysis (IPA) of EVs from control and TTLL4^plus^ cell-derived EVs. **a** Heatmap representing altered biological functions and processes in the Disease and Function category cancer based on an IPA core analysis considering statistically altered proteins (*p*-value < 0.05 or exclusively found in one condition) comparing TTLL4^plus^ EVs versus control EVs. Bar chart representation of the top three changed processes: metastasis (**b**), invasion (**c**) and neoplasia (**d**) in the Disease and Function category cancer based on IPA core analysis

To analyze whether further EV proteins may differ between control and TTLL4 overexpressing cells, proteins derived from MDA-MB231 control and TTLL^plus^ EVs were analyzed via mass spectrometric proteomics. The protein content strongly differed between control and TTLL4^plus^ cells. Of the 1270 proteins quantified in at least 3 of the 4 biological replicates per condition, 165 proteins changed significantly (*p* < 0.05) between control and TTLL4^plus^ cells (see http://www.ebi.ac.uk/pride, PXD020743). Ingenuity pathway analysis of cancer-related processes revealed that the proteins up-regulated in TTLL4^plus^ EVs mainly are involved in the control of neoplasia, metastasis and invasion (Figs. 6 and S5).

In summary, our data show that high TTLL4 expression alters the EV populations and ingenuity pathway analysis indicates that EVs derived from TTLL4^plus^ cells contain proteins involved in the regulation of malignant progression of tumour cells.

### TTLL4^plus^ cell-derived EVs alter human brain endothelial cells

Our data showing that high expression of TTLL4 significantly correlates with brain metastasis (Fig. 1d) together with the finding that EVs derived from TTLL4 overexpressing cells contain proteins involved in the control of neoplasia, metastasis and invasion (Fig. 6) led us to analyze the effect of TTLL4^plus^ derived EVs on malignancy of TNBC cells. For this, permeability of endothelial cells of the BBB (hCMEC/D3) as well as adhesion of MDA-MB231 and MDA-M468 control and TTLL4^plus^ cells to hCMEC/D3-cells were assessed.

**Fig. 7:**
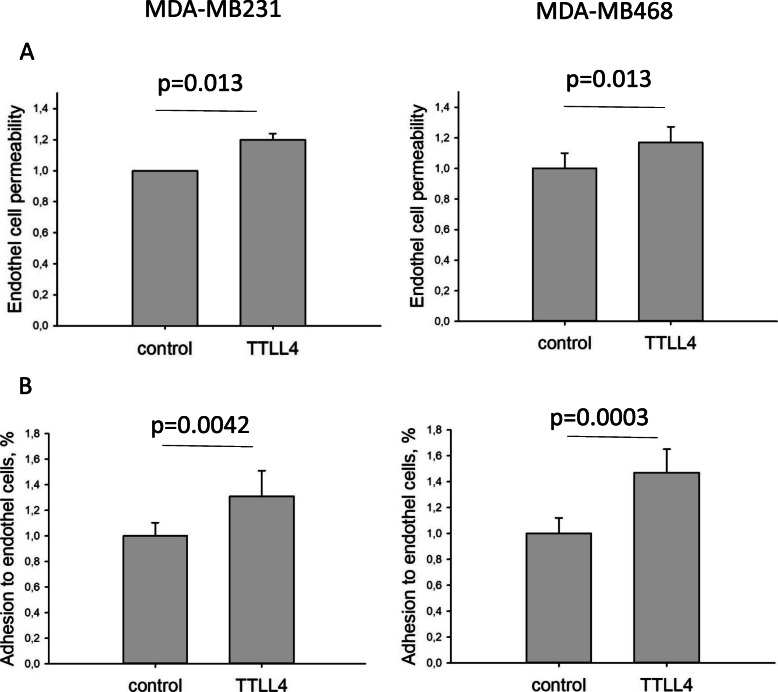
EVs from TTLL4^plus^ cells increase adhesion on and permeability of hCMEC/D3-cells. **a** hCMEC/D3-cells were grown to confluence on Transwell cell culture chambers and incubated with EVs from MDA-MB231 or MDA-MB468 control or TTLL^plus^ cells for 16 h. Thereafter, fluorescein isothiocyanate-dextran (MW 40,000) was added and, after 4 h, green fluorescence was measured in the lower reservoir. **b** hCMEC/D3-cells grown to confluence on chamber slides were incubated with EVs from MDA-MB231 or MDA-MB468 control or TTLL^plus^ cells for 16 h. Then, MDA-MB231 or MDA-MB468 control cells loaded with CellTracker™ Green CMFDA were added and after 4 h, green fluorescent cells were counted. Mean ± SD of eight different determinations are shown

Confluent hCMEC/D3-cells were treated with EVs from control or TTLL4^plus^ cells and diffusion of dextran through cell layers was measured. This analysis revealed that dextran diffusion was significantly increased by 20% in hCMEC/D3-cells pre-incubated with EVs from TTLL4^plus^ cells (Fig. 7a).

To show if also adhesion of MDA-MB231 or MDA-MB468 cells may be altered after incubating hCMEC/D3-cells with EVs derived from control or TTLL4^plus^ cells, control cells, labeled with CellTracker™ Green CMFDA, were seeded on hCMEC/D3-cells for 4 h. Quantification of labeled cells revealed that pre-incubation with TTLL4^plus^-derived EVs significantly increased adhesion of MDA-MB231 or MDA-MB468 to hCMEC/D3-cells by 31% or 47%, respectively, compared to cells incubated with EVs from control cells (Fig. 7b).

**Fig. 8:**
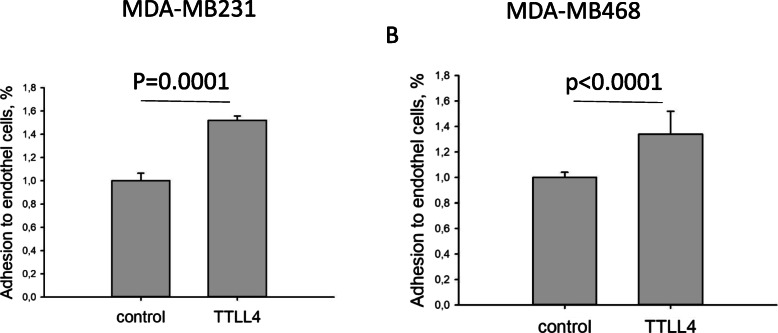
TTLL4 overexpression increases adhesion of breast cancer cells. hCMEC/D3-cells grown to confluence on chamber slides were incubated with MDA-MB231 or MDA-MB468 control cells or TTLL4^plus^ cells loaded with CellTracker™ Green CMFDA. After 4 h of incubation, green fluorescent cells were counted. Shown are mean ± SD of four different determinations

We next analyzed whether TTLL4 overexpression may also alter adhesion of breast cancer cells independent of EVs. Therefore, control or TTLL4^plus^ MDA-MB231 or MDA-MB468 cells were seeded to hCMEC/D3 cells and analyzed in the same way as above. Interestingly, we found that independent of EVs, adhesion of MDA-MB231 or MDA-MB468 TTLL4^plus^ cells was increased by 52% or 34% compared to control cells (Fig. 8).

In conclusion, these results show that up-regulation of TTLL4 in breast cancer cells promotes secretion of EV populations that increase permeability of endothelial cells of the BBB as well as tumour cell adhesion to these cells. In addition, independent of EVs, TTLL4 overexpression increases adhesion of breast cancer cells.

## Discussion

Analysis of proteins correlating with the abundance of breast cancer brain metastasis revealed that among these the only cytoskeletal-associated protein was TTLL4 (Fig. 1), a ligase that catalyzes the first addition of glutamate residue to proteins [14].

To better understand the role of TTLL4 in breast cancer cells, the protein was overexpressed in TNBC cells. Analysis of polyglutamylation of the known TTLL4 substrates NAP-1 and β-tubulin [30, 31] revealed that TTLL4 overexpression only slightly increased polyglutamylation of NAP-1, but strongly increased polyglutamylation of β-tubulin (Fig. 2b). Since also in control MDA-MB231 cells NAP-1 was strongly polyglutamylated, we assume that TTLL4 is not the main NAP-1 polyglutamylating enzyme. Alternatively, high NAP-1 polyglutamylation level could be endogenously almost saturated and therefore only slightly increasable by TTLL4 overexpression.

Our result, showing that TTLL4 overexpression mainly increases tubulin polyglutamylation has been also shown by Van Dijk et al. in in HeLa cells [31] and led us to analyze potential effects of this PTM on MT functions. Since MT-PTMs can function as “traffic lights” for intracellular transport (reviewed in 34,35), we analyzed a potential effect of TTLL4 overexpression on vesicle trafficking. The results of these analyses showed enhanced mobility of secretory vesicles and late Rab7-specific endosomes/MVBs (Fig. 3a, c). In contrast, no effect on velocity of early Rab5-specific endosomes was observed in TTLL4^plus^ cells (Fig. 3b).

Furthermore, populations of Rab7-vesicles, co- and non-colocalizing with polyglutamylated MTs were detected, suggesting that polyglutamylated MTs might transport particular Rab7-vesicles (Fig. 3d). This distinction is supported by studies reporting increased affinity [23, 24] and motility [32] of specific motor proteins interacting with mono- or short glutamylated MTs. Particularly, Kinesin-2 showed higher motility when MTs were modified with short Glu chains [32]. Furthermore, diminishing long Glu chains by knockout of TTLL1 did not affect Kinesin-2 [23]. Even the inhibitory effects of tyrosinated tubulin for Kinesin-2 binding can be overwritten by short Glu chains [24]. Recent findings have revealed that late endosomes are also transported by Kinesin-2 besides Kinesin-1 [34] to the MT-plus-ends [35–37]. However, future studies are necessary to show if TTLL4-mediated glutamylation of MTs indeed increases affinity and motility of specific kinesins and thereby trafficking of particular Rab7-vesicle populations.

The finding that TNBC cells overexpressing TTLL4 secrete EV populations different from those of control cells again supports our assumption that EV biogenesis might be affected by altered intracellular transport. According to our proteome data, these EVs from TTLL4^plus^ cells contain protein cargos increasing malignancy of cancer cells (Figs. 6 and S5B). Indeed, analysis of the biological effects of EVs revealed that the EVs derived from TTLL4^plus^ cells increased adhesion of TNBC cells to endothelial cells of the BBB (hCMEC/D3) (Fig. 7b). In addition, increased permeability of dextran through hCMEC/D3-cells was observed in presence of EVs from TTLL4^plus^ compared to control cells (Fig. 7a). These results indicate that EVs from TTLL4^plus^ cells facilitate a pre-metastatic niche to increase adhesion of breast cancer cells to the BBB. Also, elevated permeability of endothelial cells treated with EVs from TTLL4^plus^ cells may facilitate invasion of breast cancer cells through the BBB. This hypothesis is supported by our proteome data, showing increased levels of proteins involved in invasion in EVs from TTLL4^plus^ cells (Figs. 6 and S5B). However, since we did not provide direct evidence that TTLL4^plus^ cell-derived EVs increase invasion of breast cancer cells through the endothelial cell layer, this potential activity must be analyzed in detail in future studies. In addition, the specific molecular changes induced by TTLL4^plus^ cell-derived EVs on endothelial cells are still unknown. Tominaga et al. 2015 [23] found that cancer-derived EVs increased permeability of endothelial tight junctions by tranfer of miR-181c to endothelial cells. Furthermore, an exosome-mediated enhanced expression of intercellular adhesion molecule 1 (ICAM-1) and vascular cell adhesion molecule 1 (VCAM-1) in human endothelial cells have been described in a model for chronic myelogenous leukemia [38]. Interestingly, our data also showed an increased ICAM-1 concentration in EVs derived from TTLL4^plus^ cells (Figure S5B). Future studies will show if this ICAM-1 up-regulation is involved in the EV-facilitated adhesion of TNBC cells to hCMEC/D3 cells.

MT-PTMs affect malignancy of tumour cells not only by modulating EV signatures but also by directly changing cellular MT functions. Whipple et al. [39] revealed that increased MT-stability in breast cancer cells, mediated by detyrosination of the C-terminal glutamate, facilitates formation of microtentacles, enhancing attachment to endothelial cells. This finding is in accordance with our result, showing that independent of EVs, TTLL4 overexpression increases adhesion of MDA-MB231 and MDA-MB468 to hCMEC/D3 cells (Fig. 8). Thus, it seems that MT-PTMs directly control cellular behaviour and provide a metastatic niche by altering EV signature.

In summary, here we demonstrate that TTLL4 was the only cytoskeleton-associated protein whose up-regulation in breast cancer cells correlated with the formation of brain metastasis. Functional studies suggest that TTLL4-mediated glutamylation of β-tubulin increases trafficking of MVBs and results in altered EV signature. This EV signature increases adhesion of breast cancer cells to endothelial cells of the BBB as well as permeability of these endothelial cells.

## Conclusions

Here we show for the first time that TTLL4-overexpression enhances MVB motility, thus altering EV homeostasis. Moreover, we reveal that EVs secreted by TTLL4^plus^ cells increase the potential of breast cancer cells to adhere to endothelial cells of the BBB. These findings support our clinical data, showing that high TTLL4 expression correlates with brain metastasis of breast cancers. Hence, TTLL4 may be an interesting target for therapy. Our work provides a basis for future research, including pre- and clinical investigations examining the effectors and biogenesis of TTLL4-altered exosomes.

## Supplementary information

**Additional file 1: Figure S1.** Clinical relevance of TTLL4 levels for breast cancer patients. Kaplan-Meier analysis show TTLL4 mRNA levels of all four quartiles for overall survival and shorter recurrence-free survival. A high correlation is shown by *p*-values after log-rank tests comparing two groups (TTLL4 levels < 75% vs. TTLL4 levels > 75%). (C-E). Significant associations of high TTLL4 levels with negative estrogen (ER) levels (C), negative progesterone (PR) levels (D), and lung metastasis formation (E) are shown. *P*-values after χ^2^-Test are given. **Figure S2.** TTLL4 mRNA expression of different breast cancer cells lines. mRNA expression of TTLL4 was analyzed by real-time PCR and normalized to the value of MDA-MB231 cells. MDA-MB231-BR = cells that preferentially metastasize to the brain. MDA-MB231-SA = cells that preferentially metastasize to the bone. **Figure S3.** Effect of TTLL4 on actin dynamics. (A) F-actin concentration of phalloidin-stained cells. (B, D). Cellular protrusions were stained by AF-488 coupled phalloidin (B) or by an AF-488 coupled antibody against VASP (C). Mean values ± SD of three different experiments are shown. **Figure S4.** Overexpression of TTLL4 in MDA-MB468 cells. (A) TTLL4 mRNA levels of MDA-MB231 and MDA-MB468 control cells, normalized to TTLL4 mRNA from MDA-MB231 cells. (B) TTLL4 mRNA levels of MDA-MB468 control cells and cells treated with a lentiviral vector coding for TTLL4, normalized to TTLL4 mRNA from control MDA-MB468 cells. (C) Protein lysates from control and TTLL4 overexpressing MDA468 cells were analyzed for polyglutamylation by Western blotting using the GT335 antibody. Detection of β-tubulin and Hsc70 served as loading control. **Figure S5.** Concentration and volcano plot of proteins regulated in EVs derived from TTLL4 overexpressing cells. (A) Protein concentration of EVs was determined by the Bradford assay and particles/ml by NTA. From the resulting values, μg/1*10 ^10^ particles were calculated. Shown are mean values ± SD of four different determinations. (B) EVs proteins from control or TTLL4 overexpressing cells were analyzed by mass spectrometry and the log ratios of TTLL4 vs. control proteins were calculated.

**Additional file 2.** Supplementary methods.

## Data Availability

All data generated or analysed during this study are included in this published article and its Supplementary files.

## Abbreviations

HEPES: 4-(2-hydroxyethyl)-1-piperazineethanesulfonic acid
DAPI: 4′,6-Diamidin-2-phenylindol
CMFDA: 5-chloromethylfluorescein diacetate
AF: Alexa-Fluor
BBB: Blood-brain-barrier
BDNF: Brain-derived neurotrophic factor
BC: Breast cancer
cDNA: Complementary DNA
DNA: Deoxyribonucleic acid
DMSO: Dimethylsulfoxide
ER: Estrogen receptor
EDTA: Ethylenediaminetetraacetic acid
FCS: Fetal calf serum
F-actin: Filamentous actin
Glu: Glutamate
GA: Glutaraldehyde
GFP: Green fluorescent protein
HER2: Human epidermal growth factor receptor 2
ICAM-1: Intercellular adhesion molecule 1
ILV: Intraluminal vesicles
LFQ: Label-free quantification
LoG detector: Laplacian of Gaussian particle detector
Lap Tracker: Linear assignment problem tracker
LC-MS/MS: Liquid chromatography-mass spectrometry/mass spectrometry
mRNA: Messenger RNA
MTT: Methylthiazolyldiphenyl-tetrazolium bromide
miRNA: Micro ribonucleic acid
MT: Microtubule
MAP: Microtubule-associated proteins
MDA-MB: Monroe Dunaway Anderson Metastatic Breast cancer cells
MVB: Multivesicular bodies
mRFP: Monomeric red fluorescent protein
NTA: Nanoparticle tracking analysis
ncRNA: Non-coding ribonucleic acid
NAP: Nucleosome assembly protein
PFA: Paraformaldehyde
PBS: Phosphate-buffered saline
PTM: Posttranslational modifications
PR: Progesterone receptor
Rab5: Ras-related protein 5
Rab7: Ras-related protein 7
rt-PCR: Real-time polymerase chain reaction
RT: Room temperature
SD: Standard deviation
TC: Tissue culture
TEM: Transmission electron microscopy
TNBC: Triple-negative breast cancer cells
TTLL4^plus^: Tubulin Tyrosine Ligase Like 4 overexpression
TTLL: Tubulin Tyrosine Ligase Like
UKE: University Medical Center Hamburg-Eppendorf
VCAM-1: Vascular cell adhesion molecule 1
VE-Cadherin: Vascular endothelial cadherin
VASP: Vasodilator-stimulated phosphoprotein
