# Supplementary material for: Tubulin Tyrosine Ligase Like 4 (TTLL4) overexpression in breast cancer cells is associated with brain metastasis and alters exosome biogenesis
Source: J Exp Clin Cancer Res. 2020 Sep 30;39:205. doi: 10.1186/s13046-020-01712-w (PMC7528497; doi:10.1186/s13046-020-01712-w)
Supplement: Supplementary file 1 — Additional file 1: Figure S1. Clinical relevance of TTLL4 levels for breast cancer patients. Kaplan-Meier analysis show TTLL4 mRNA levels of all four quartiles for overall survival and shorter recurrence-free survival. A high correlation is shown by p-values after log-rank tests comparing two groups (TTLL4 levels < 75% vs. TTLL4 levels > 75%). (C-E). Significant associations of high TTLL4 levels with negative estrogen (ER) levels (C), negative progesterone (PR) levels (D), and lung metastasis formation (E) are shown. P-values after χ2-Test are given. Figure S2. TTLL4 mRNA expression of different breast cancer cells lines. mRNA expression of TTLL4 was analyzed by real-time PCR and normalized to the value of MDA-MB231 cells. MDA-MB231-BR = cells that preferentially metastasize to the brain. MDA-MB231-SA = cells that preferentially metastasize to the bone. Figure S3. Effect of TTLL4 on actin dynamics. (A) F-actin concentration of phalloidin-stained cells. (B, D). Cellular protrusions were stained by AF-488 coupled phalloidin (B) or by an AF-488 coupled antibody against VASP (C). Mean values ± SD of three different experiments are shown. Figure S4. Overexpression of TTLL4 in MDA-MB468 cells. (A) TTLL4 mRNA levels of MDA-MB231 and MDA-MB468 control cells, normalized to TTLL4 mRNA from MDA-MB231 cells. (B) TTLL4 mRNA levels of MDA-MB468 control cells and cells treated with a lentiviral vector coding for TTLL4, normalized to TTLL4 mRNA from control MDA-MB468 cells. (C) Protein lysates from control and TTLL4 overexpressing MDA468 cells were analyzed for polyglutamylation by Western blotting using the GT335 antibody. Detection of β-tubulin and Hsc70 served as loading control. Figure S5. Concentration and volcano plot of proteins regulated in EVs derived from TTLL4 overexpressing cells. (A) Protein concentration of EVs was determined by the Bradford assay and particles/ml by NTA. From the resulting values, μg/1*10 10 particles were calculated. Shown are mean values ± SD of fo [file 13046_2020_1712_MOESM1_ESM.pptx]

## Slide 1
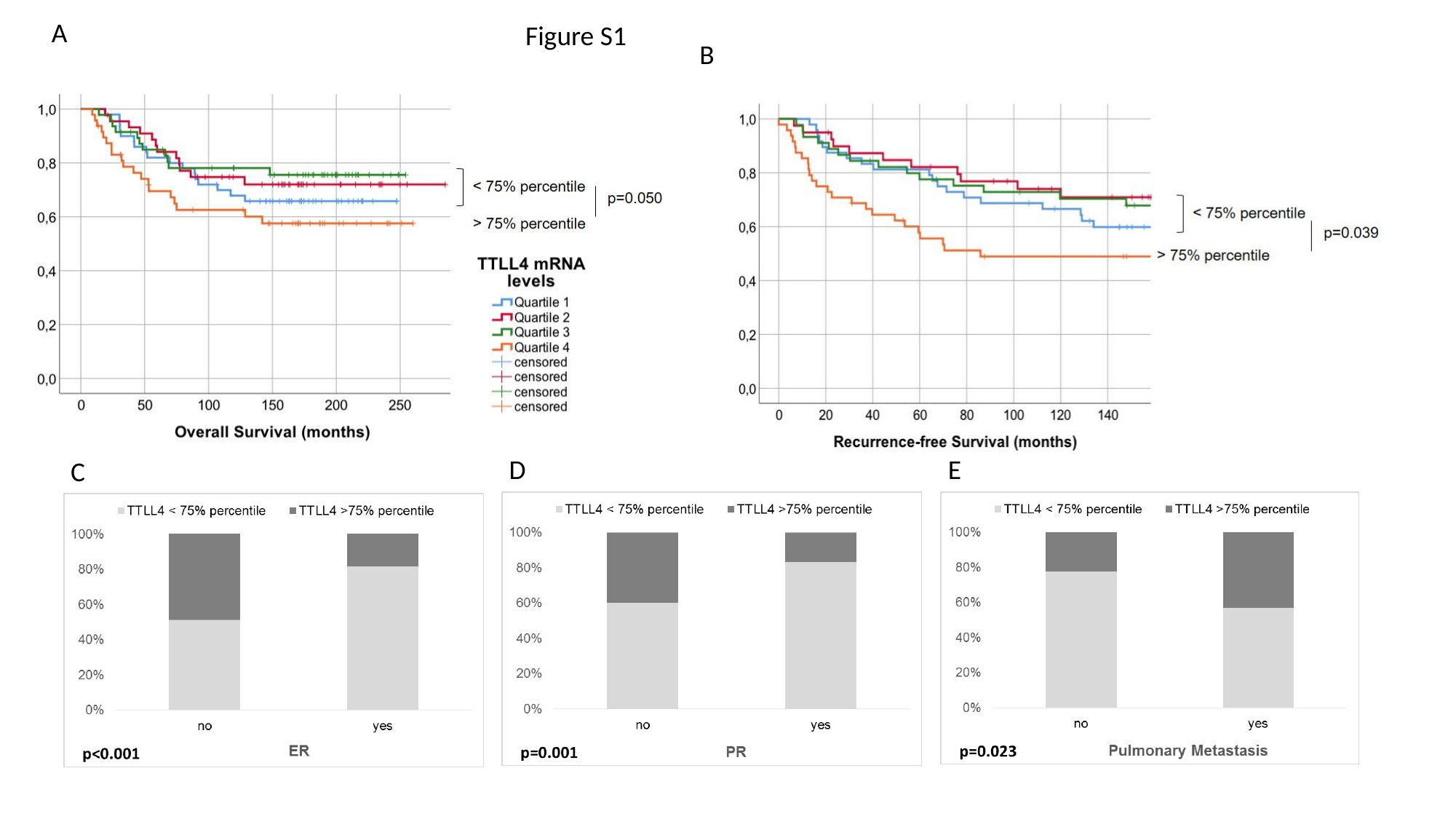

A
Figure S1
B
E
D
C

## Slide 2
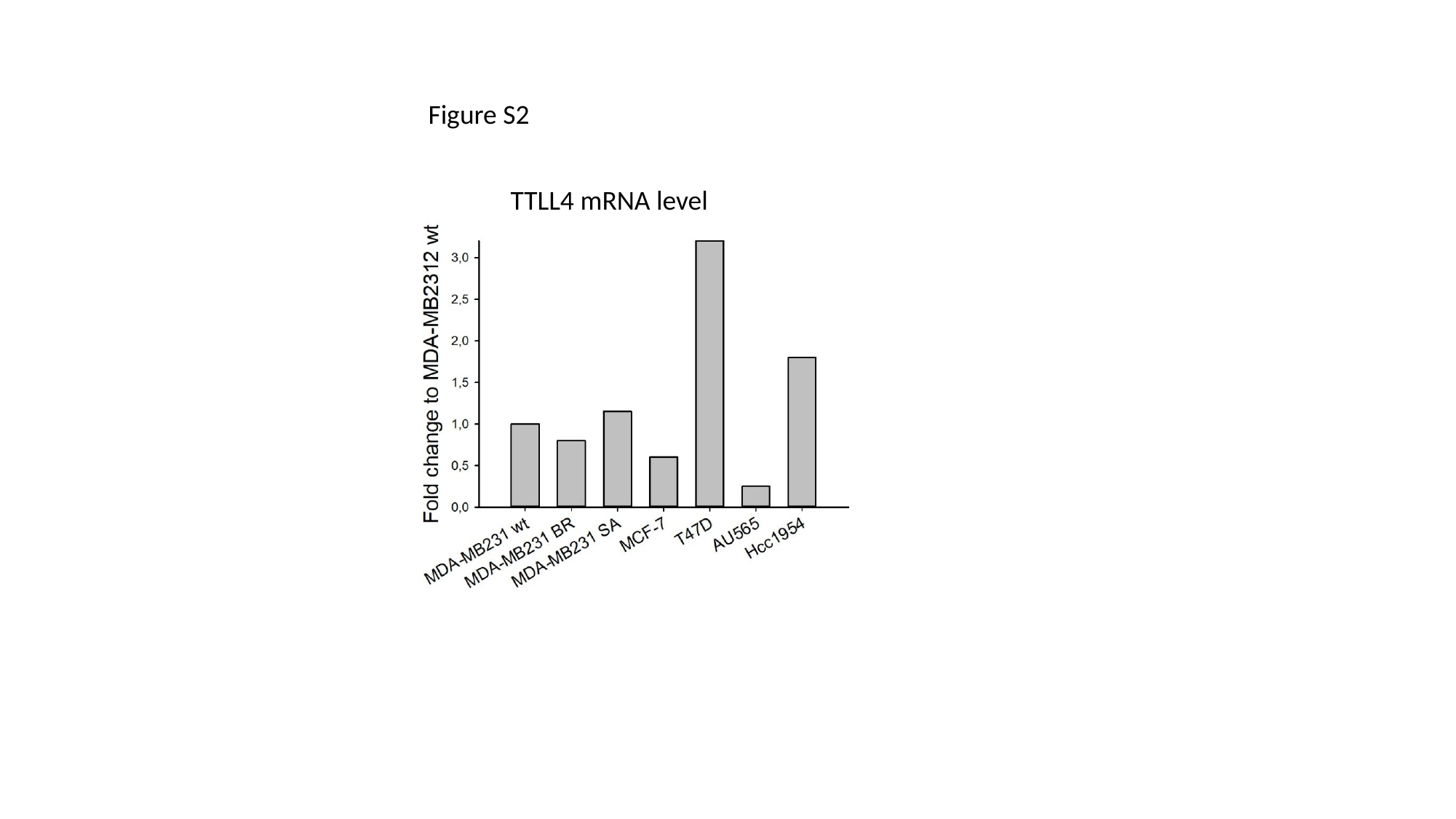

Figure S2
TTLL4 mRNA level

## Slide 3
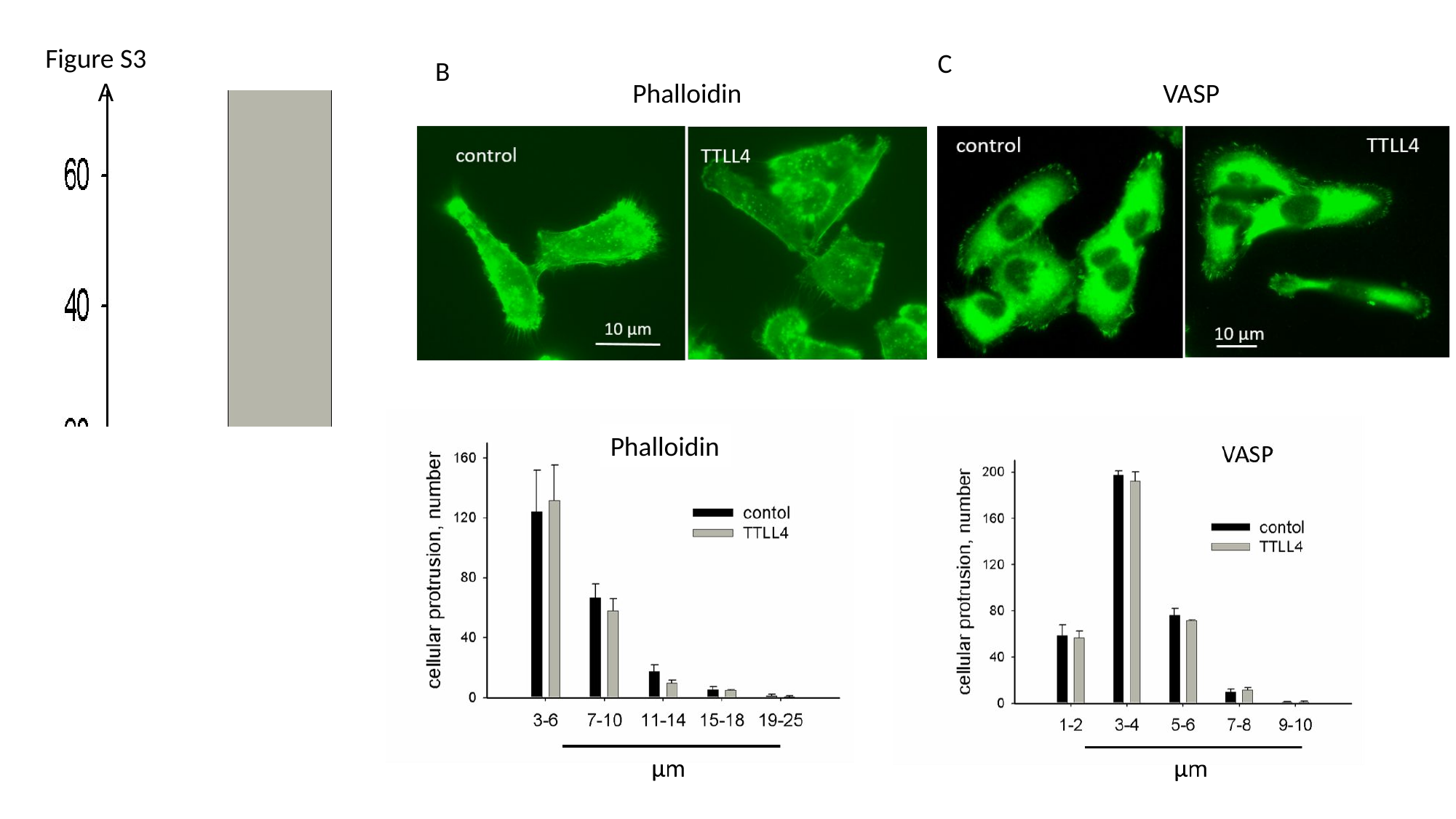

Figure S3
C
B
A
Phalloidin
VASP
Phalloidin

## Slide 4
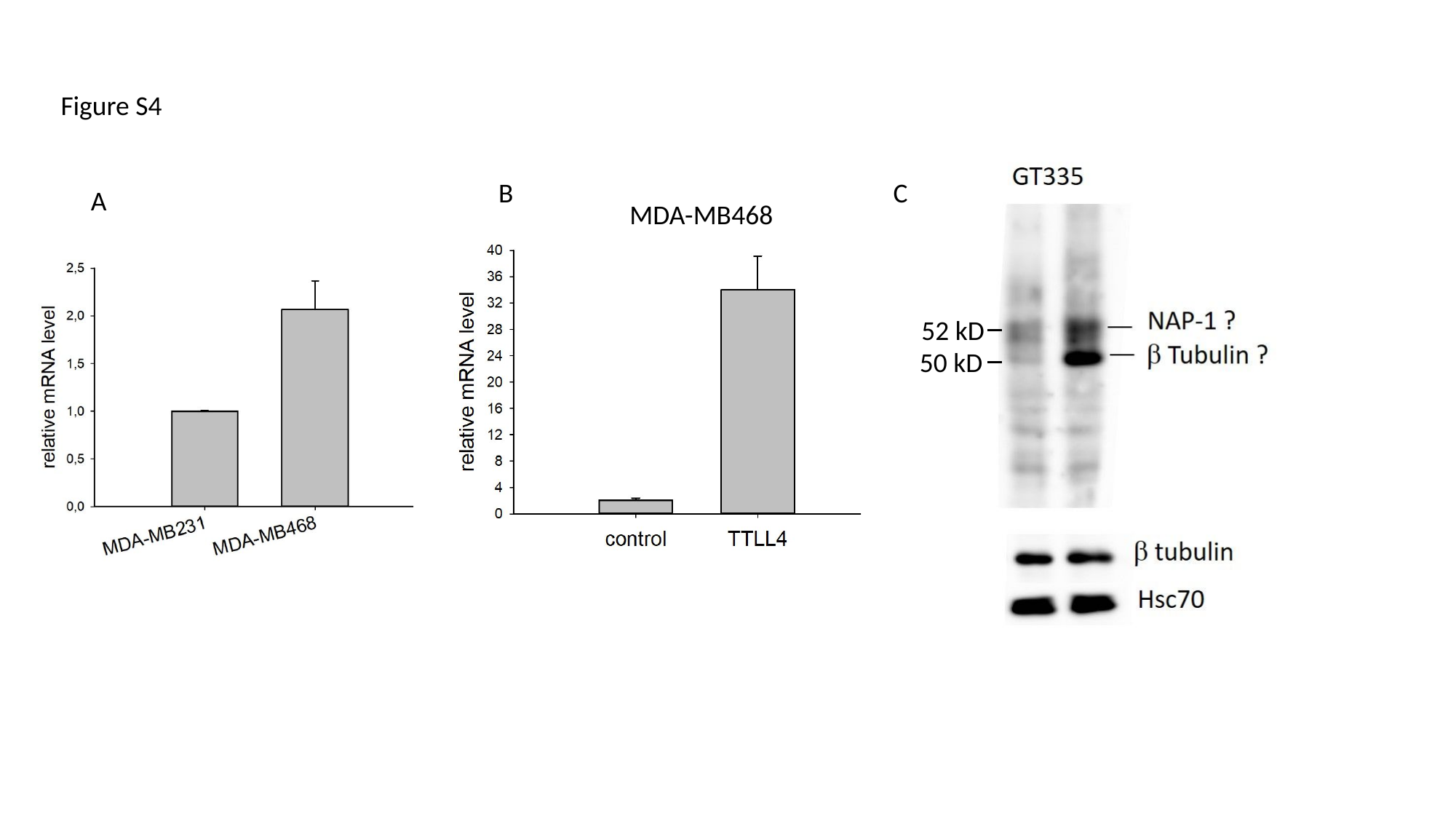

Figure S4
B
C
A
MDA-MB468
52 kD
50 kD

## Slide 5
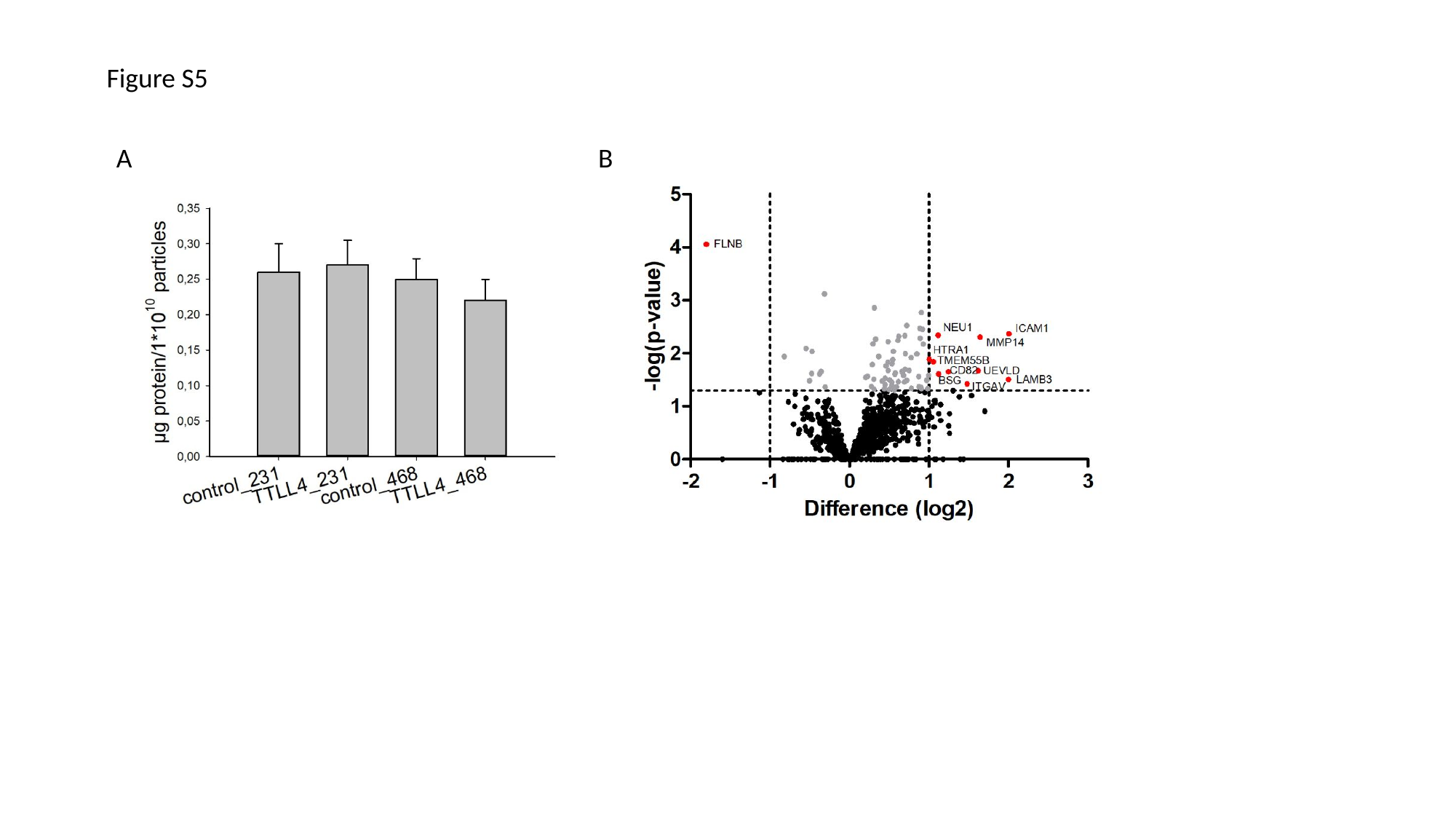

Figure S5
A
B
