# Supplementary material for: Tubulin Tyrosine Ligase Like 4 (TTLL4) overexpression in breast cancer cells is associated with brain metastasis and alters exosome biogenesis
Source: J Exp Clin Cancer Res. 2020 Sep 30;39:205. doi: 10.1186/s13046-020-01712-w (PMC7528497; doi:10.1186/s13046-020-01712-w)
Supplement: Supplementary file 2 — Additional file 2. Supplementary methods. [file 13046_2020_1712_MOESM2_ESM.docx]

**Supplementary methods**

**Detailed methods for mass spectrometry of EVs.** Exosome samples were lysed in 100 mM ammonium bicarbonate (Thermo Fisher) and 1% w/v sodium deoxycholate (SDC, Sigma Aldrich) buffer boiled at 95 °C for 5 min and sonicated with a probe sonicator to destroy DNA/RNA. 10 μg of protein was used and reduced in presence of 10 mM dithiothreitol (DTT, Sigma Aldrich) at 60 °C for 30 min and alkylated with 20 mM iodoacetamide (IAA, Sigma Aldrich) for 30 min in the dark at 37 °C. Trypsin (sequencing grade, Promega) was added at a 1:100 ratio (enzyme to protein) and digestion was performed overnight at 37 °C. To stop the reaction and precipitate the SDC, formic acid (FA, Fluka) was added to 1% final concentration. The samples were centrifuged for 5 min at 16.000 g, the supernatant was transferred into a new tube and was dried in a vacuum centrifuge. For LC-MS/MS analyses, samples were resuspended in 0.1% FA at a concentration of 1 μg/μl. LC-MS/MS measurements were done on a Quadrupole Orbitrap hybrid mass spectrometer (Q Exactive, Thermo Fisher) coupled to a UPLC system (nanoAcquity, Waters). For analysis, 1 μg of peptides were loaded by autosampler injection onto a C18 reversed-phase (RP) trap column (Symmetry C18 trap column, 100 Å pore size, 5 μm particle diameters, 180 μm x 20 mm) and separated on a 25 cm C18 RP (Peptide BEH C18 column, 130 Å pore size, 1.7 μm particle diameters, 75 μm x 250 mm). Trapping was done for 5 min at a flow rate of 15 μl/min with 99% solvent A (0.1% FA) and 1% solvent B (0.1% FA in ACN). Separation and elution of peptides were achieved by a linear gradient from 1 to 30% solvent B in 60 min. The eluting peptides were transferred in an Orbitrap Q Exactive mass spectrometer. MS1 scans were performed in positive mode over a scan range of 400-1300 m/z. The Orbitrap resolution was set to 70.000 with an AGC target of 1x10^6^ and a maximum injection time of 240 ms. Peptides with charge states between 2+ - 5+ above an intensity threshold of 100.000 were isolated with a 2 m/z isolation window in Top12 mode and fragmented with a normalized collision energy of 28%. The fragments were measured with an Orbitrap resolution of 17.500, AGC target of 1x10^5^ and 50 ms maximum injection time. Already fragmented peptides were excluded for 20 seconds. The collected raw files were searched against the reviewed human protein sequence database (Uniprot, EMBL, release April 2018 with 20260 entries) and the reviewed bovine protein sequence database (Uniprot, EMBL, release June 2020, 6012 entries). They were processed with the Andromeda Algorithm included in the MaxQuant Software (Max Plank Institute for Biochemistry, Version 1.6.2.10). All samples were handled as individual experiments and the label-free quantification option with a match between runs was used. Trypsin was selected as enzyme used to generate peptides, allowing a maximum of two missed cleavages. A minimal peptide length of 6 amino acids and maximal peptide mass of 6000 Da was defined. Oxidation of methionine, acetylation of protein N-termini and the conversion of glutamine to pyro-glutamic acid were set as variable modification. The carbamidomethylation of cysteines was selected as fixed modification. The error tolerance for the first precursor search was 20 ppm, for the following main search 4.5 ppm. Fragment spectra were matched with 20 ppm error tolerance. The false discovery rate for peptide spectrum matches and proteins were set to 1%. For quantification, all identified razor and unique peptides were considered. The ProteinGroups.txt result file from MaxQuant was filtered for proteins derived from human origin or proteins showing more or equal numbers of unique peptides from human and bovine origin. Proteins only derived from bovine origin or with a higher number of unique peptides from bovine origin were removed. The reduced ProteinGroup.txt file was loaded into Perseus software (Max Plank Institute for Biochemistry, Version 1.5.8.5). To perform data normalization and statistical testing, Student’s T-Test was used. Proteins present in at least three of four biological replicates in at least one condition were considered for the quantitative analysis. Considered proteins were uploaded together with *p*-value and fold change information (proteins exclusively found in one condition were included with either positive or negative infinite values) into ingenuity pathway analysis software (QIAGEN) and a core analysis was performed.
